# Safety and Feasibility of a Novel Protocol for Percutaneous Dilatational Tracheostomy in Patients with Respiratory Failure due to COVID-19 Infection: A Single Center Experience

**DOI:** 10.1155/2021/8815925

**Published:** 2021-01-13

**Authors:** Ziad Boujaoude, Nagendra Madisi, Bhavi Patel, Jean-Sebastien Rachoin, R. Phillip Dellinger, Wissam Abouzgheib

**Affiliations:** ^1^Division of Pulmonary and Interventional Pulmonary Medicine, Department of Medicine, Cooper University Health Care, Cooper Medical School of Rowan University, Camden, NJ, USA; ^2^Division of Critical Care Medicine, Department of Medicine, Cooper University Health Care, Cooper Medical School of Rowan University, Camden, NJ, USA

## Abstract

**Introduction:**

The rapidly spreading Novel Coronavirus 2019 (COVID-19) appeared to be a highly transmissible pathogen in healthcare environments and had resulted in a significant number of patients with respiratory failure requiring tracheostomy, an aerosol-generating procedure that places healthcare workers at high risk of contracting the infection. Instead of deferring or delaying the procedure, we developed and implemented a novel percutaneous dilatational tracheostomy (PDT) protocol aimed at minimizing the risk of transmission while maintaining favorable procedural outcome. *Patients and Methods*. All patients who underwent PDT per novel protocol were included in the study. The key element of the protocol was the use of apnea during the critical part of the insertion and upon any opening of the ventilator circuit. This was coupled with the use of enhanced personnel protection equipment (PPE) with a powered air-purifying respirator (PAPR). The operators underwent antibody serology testing and were evaluated for COVID-19 symptoms two weeks from the last procedure included in the study.

**Results:**

Between March 12th and June 30th, 2020, a total of 32 patients underwent PDT per novel protocol. The majority (80%) were positive for COVID-19 at the time of the procedure. The success rate was 94%. Only one patient developed minor self-limited bleeding. None of the proceduralists developed positive serology or any symptoms compatible with COVID-19 infection.

**Conclusion:**

A novel protocol that uses periods of apnea during opening of the ventilator circuit along with PAPR-enhanced PPE for PDT on COVID-19 patients appears to be effective and safe for patients and healthcare providers.

## 1. Introduction

Since it was first identified in Wuhan City, Hubei Province, China, on December 30th, 2019, the Novel Coronavirus 2019 (COVID-19) has spread rapidly and widely resulting in a worldwide pandemic, affecting over 22 million persons worldwide and causing over 700 thousand deaths to date [1]. While the majority of infected patients do not develop severe disease, around 15% may develop significant dyspnea and hypoxemia and 5% may evolve to respiratory failure, acute respiratory distress syndrome (ARDS), and/or multiorgan failure that require invasive ventilatory support for an extended period of time [2, 3]. Tracheostomy is a common procedure for those critically ill patients, but it is an aerosol-generating procedure that places healthcare workers (HCW) at risk of infection during insertion and subsequent care. Similar to the severe acute respiratory syndrome (SARS) pandemic in 2003, COVID-19 appeared to be a highly transmissible pathogen in healthcare environments [4].

At the beginning of the current pandemic, medical societies and experts have recommended to delay or avoid tracheostomies in this population. The American Academy of Otolaryngology and the Ears, Nose, and Throat Surgery in the United Kingdom, have stated that providers should “avoid tracheotomy in COVID-19 positive or suspected patients” due to the risks to healthcare providers. They recommended that tracheostomies should not be performed before 2-3 weeks after intubation, preferably after negative COVID-19 testing, and favored open tracheostomy placement in these circumstances as opposed to percutaneous dilatational tracheostomy (PDT) [5, 6]. Earlier tracheostomy, however, would assist in resource preservation by facilitating weaning from mechanical ventilation as well as throughput to a lower level of care [7]. PDT is routinely performed in our medical ICU by the interventional pulmonary service. This emerging situation presented us with a new challenge of maintaining a balance between providing medical care to those in need while limiting disease spread and exposure to patients and staff. The decision was made at our institution to not delay tracheostomy based on the presence of COVID-19 but instead to establish a protocol that would facilitate safe PDT. We developed and implemented a novel protocol that introduced several modifications to the standard technique designed to minimize the risk of aerosolization of the virus while maintaining a low risk of complications. We report in this study the feasibility of this novel protocol and its safety for the patients and the providers.

## 2. Patients and Methods

### 2.1. Patient Population

We collected data on all patients admitted to the intensive care unit (ICU) at Cooper University Hospital (CUH), from March 12th to June 30th, 2020, who had confirmed COVID-19 by nasal pharyngeal swab for reverse transcriptase polymerase chain reaction (rtPCR) assay, who required mechanical ventilation for severe respiratory failure, and who underwent PDT per novel protocol by the interventional pulmonary service. The CUH institutional review board approved this study (IRB #120-00475).

### 2.2. Novel Percutaneous Tracheostomy Protocol

PDT was performed at the bedside in the patient's ICU room. Timing of the procedure should be at least 2 weeks from time of intubation in line with our standard procedure. A negative PCR was preferable but not necessary. The team participants were limited to only essential personnel—this included one of the two hospital interventional pulmonologist, one of the two interventional pulmonary fellows in training, a registered nurse, and a respiratory therapist. A technician and nearby nurses were available outside the room to obtain equipment or medications as needed. All equipment and medications were checked prior to entering the room and then were taken into the room, including an extra supply of medication that potentially may be used. Backup equipment was left immediately outside the room with a technologist to be readily available if needed. A powered air-purifying respirator (PAPR) with standard donning (per CDC guidelines) was utilized by all four members of the team along with an N95 mask under the PAPR hood as a backup in case of an unexpected malfunction [8]. Full sedation and analgesia were used as well as neuromuscular blockade, to prevent cough and minimize aerosolization.

Mechanical ventilation setting thresholds recommended to proceed with PDT were a positive end expiratory pressure (PEEP) ≤ 12 mmHg and a fraction of inspired oxygen (FIO_2_) ≤ 60%. A 60-second apnea test on 100% FIO_2_ was performed to assess the patient's ability to tolerate apnea during the critical part of the procedure. The ventilator was placed on standby while the patient's oxygen saturations and hemodynamics were monitored for 60 seconds. If at any point during the apnea the patient experienced oxygen desaturation below 90% and or hemodynamic instability, the apnea test was aborted, and the procedure deferred. After passing the apnea test, the patient was fully draped in sterile fashion to avoid any contamination of equipment and bed. Ventilation was paused for the introduction of a bronchoscope into the endotracheal tube (ETT).

A single-use flexible Ambu® aScope™ (Ambu A/S, Ballerup, Denmark) was used. Once the bronchoscope was introduced, ventilation was resumed. The bronchoscopist and the respiratory therapist retracted the endotracheal tube (ETT) in harmony, while maintaining an adequate intraluminal view of the trachea until ETT was just proximal to the 1st tracheal ring. The cuff of the ETT remained inflated to minimize any aerosolization. Then, the main operator proceeded with incision and needle insertion between the first and second tracheal ring under direct visualization via bronchoscope. Of note, the syringe attached to the needle was not removed immediately. Ventilation was then paused, at which point the rest of the procedure was performed under the standard PDT technique. Once tracheostomy was inserted and visualized with the bronchoscope from above through the ETT, the cuff was inflated, the inner cannula was immediately inserted, the ventilator was attached via flexible tubing, and ventilation was resumed. EtCO_2_ was not checked, but inspiratory and expiratory volumes were observed on the ventilator. (Table 1).

### 2.3. End Points

The endpoints for this study were the safety and feasibility of our PDT protocol, the healthcare providers' contraction of COVID-19 virus (development of COVID-19 symptoms and/or positive serology testing), and posttracheostomy early patients' outcome. The constant members of the tracheostomy team included the four physicians. They all agreed to testing with COVID-19 antibody serology (IgG and IgM against SARS-CoV-2) and an evaluation for COVID-19 symptoms fourteen days from the last procedure included in the study. The other members were variable and included different nurses and respiratory therapists working in the ICU. Individual evaluation for the variable members was not possible.

## 3. Results (Table 2)

Between March 12th and June 30th, 2020, a total of 32 patients underwent PDT using the novel protocol. The majority (80%) were PCR positive at the time of procedure. The mean age was 54 ± 12 years; 50% were male; the mean body mass index (BMI) was 33 ± 10. At the time of tracheostomy, all patients had FIO_2_ and PEEP less than 60% and 12, respectively, with mean FIO_2_ of 44 ± 8 and PEEP of 8 ± 3. The procedure was successful in 30 of 32 patients (94%). All patients passed the apnea test. In two patients, there was variation from the set protocol. In one patient, desaturation occurred after needle insertion; the ET tube was then advanced and positioned above the carina with dilation and insertion performed next to the ETT. In another patient, the flow was restarted after needle insertion due to severe desaturation. There were no direct complications related to the tracheostomy procedure. Only an early onset minor bleeding was present in one patient and self-resolved.

### 3.1. Healthcare Providers' Outcome

None of the four proceduralists developed symptoms suggestive of COVID-19 infection and none tested positive for COVID-19 antibodies. None have missed any days of work and to this date all continue to perform their usual clinical duties.

### 3.2. Early Patients' Outcome

The mean time from the start of invasive mechanical ventilation to PDT was 22 ± 8 days. The mean follow-up for all the patients after starting mechanical ventilation was 40 ± 14 days, and after PDT, it was 17 ± 10 days. Of the 32 patients who underwent a PDT, 18 patients were weaned from MV (56%), 5 remained on full ventilator support (16%), and 9 died as a result of respiratory and/or multiorgan failure (28%).

## 4. Discussion

We describe in this retrospective study a novel protocol for percutaneous placement of tracheostomy in patients with respiratory failure due to COVID-19 infection. It was designed to minimize the risk of healthcare workers' aerosol exposure while maintaining optimal procedural safety and outcome. To our knowledge, this study is the first to report on operator's safe outcome for COVID-19 contraction during tracheostomy. We have demonstrated the protocol to be safe and effective with no negative impact on patients or staff.

Tracheostomy is a major aerosol-generating procedure that was identified as a leading cause of viral transmission and superspreading events during the SARS outbreak in 2003 [5]. A systematic review of 10 studies from the 2003 SARS outbreak suggests that tracheostomy has an OR of 4.2 for risk of transmission to healthcare workers (HCWs) [9]. While severe acute respiratory syndrome coronavirus 2 (SARS-CoV-2) is associated with lower mortality than the related viruses that cause severe acute SARS and Middle East respiratory syndrome (MERS), it appears to have a higher infectivity and rates of transmission [10]. An early report of infections related to aerosol-generating procedures has already emerged in the current pandemic [11].

When the influx of COVID-19 critically ill patients admitted to our ICUs began to accelerate in early March, the available data regarding guidance on how to mitigate the risk of transmission was very limited [10, 12–14]. We were faced with the need to rapidly develop and implement a PDT protocol aimed at minimizing the risk of aerosol exposure to the staff while maintaining safe and effective care to the patients in need.

Tracheostomy procedures are performed at bedside in the ICU to minimize the risk of transmission during transport. Since not all rooms are negative pressure flow rooms, enhanced PPE with PAPR was used by all members of the team.

The main focus of the protocol was on maintaining a closed ventilator circuit at all times and opening only during periods of apnea. A major advantage of our protocol compared with a standard bedside PDT is the avoidance of an open-air system that leads to active ventilation/aerosolization through most of the procedure. Equally important was the use of standard timing for the procedure, a short procedural time, adequate sedation and paralytics, and the use of a disposable scope.

The protocol allows the cuff to remain inflated during the ETT retraction into the subglottic area and the ability to place the ventilator on standby when adding the adapter to the circuit, inserting the scope in the ETT and during the insertion of tracheostomy. The protocol required patient qualification characteristics of a PEEP lower than 12 cm H_2_O and an FIO_2_ lower than 60%. A negative apnea test (absence of desaturations or hemodynamic instability while ventilator is placed on standby for one minute) was also required in order to consider if the patient is able to tolerate the apnea during the procedure. The cut-offs for PEEP and FIO_2_ are similar to those that we use for non-COVID patients in our practice. The duration of the apnea test was arbitrary. Based on our experience, it does not take more than 60 seconds for an experienced operator to insert the tracheostomy from the time of successful needle insertion between the tracheal rings. The combination of PEEP/FIO_2_ cut-off of 12 cm H_2_O/60% and a negative apnea test appears to be a good predictor of apnea tolerance.

In order to minimize apnea time, all steps up to the insertion of the needle in the airway were performed before scope insertion. Identifying the landmarks, skin incision, and insertion of the needle were performed by the most experienced operator, and the apnea was not initiated until precise insertion of needle was confirmed.

A conventional timing for tracheostomy of 2 weeks from time of intubation for patients with ongoing mechanical ventilation was adopted for the protocol. Although early tracheostomy has clear benefits, including lower sedation requirements and increased patient comfort [15], the data regarding the optimum timing of tracheostomy is conflicting [16, 17] and published literature does not clearly support a mortality benefit or reduction of ventilator-associated pneumonia with earlier tracheostomy [18]. Our standard ICU general criteria based on days of mechanical ventilation is 14 days with the caveat that the patient is not improving as to weaning capability. Given that published reports from China and Italy demonstrated that COVID-19 has a high ICU mortality [19], it seemed prudent to wait until the course of disease is declared before committing patients to earlier tracheostomy and leading to procedures in patients who may not need it altogether during the course of their illness. Two weeks seemed an appropriate timing in that light.

A negative PCR test was not required as the results of the test would not impact the decision on protective measures. The test has variable sensitivity in clinical practice ranging from 37% to 71% [20]. The immune response (antiviral antibody) is more reliable in predicting infectivity, but serology testing was not available. Recent data also shows that patients may remain PCR positive despite no longer having viable virus and capability to transmit [21].

Guidance with bronchoscopy is routinely used by interventional pulmonologists during PDT, and the procedure has been demonstrated to be safe and effective. We restricted the use to disposable bronchoscopes. It allows a lower number of support staff and does not require reprocessing. This minimizes both the staff exposed and the surfaces needed to be disinfected post procedure in addition to preservation of critical PPE. Of note, bronchoscopy has been commonly required in critically ill patients with COVID-19 to manage complications like atelectasis and hemoptysis, particularly for patients on ECMO, and to obtain samples for microbiological cultures [22, 23]. We favor the use of a disposable bronchoscope for these indications.

A key finding of this study is that all four physicians providing the procedure were COVID-19 negative by symptoms and/or by serology 14 days after the last procedure included in the study. The sensitivity of serologic testing since the time of infection is very high, and the rate of positive IgG approaches 100 percent at 19 days [24]. All physicians wore enhanced PPE during the procedure. Although the use of PPE and PAPR has been reported to limit communication (hearing) among members of the team, no issues were reported as a consequence of wearing PPE [25]. While the availability of PAPR may represent a potential limitation to implementing our protocol, given the limited number of PAPR used and the nonurgent nature of the procedure, we believe that this will not be an issue for most intensive care units.

The use of apnea during tracheostomy is not new. A recent report by Niroula et al. described the use of apnea during tracheostomy on COVID patients [26]. They concluded that the technique is safe, but they did not report on healthcare workers' safety. To our knowledge, our study is the first to report on operators' safe outcome for COVID-19 contraction. Furthermore, their apnea duration was significantly longer with an average of 3.96 min versus less than one minute in our study. This is due to a major difference between protocols. We did not initiate apnea until we confirmed adequate insertion of the needle. A shorter time of ventilation disruption provide a major advantage for patients with limited respiratory reserve in terms of ability to tolerate the procedure.

The main limitation of this study is that not all healthcare workers involved in the procedure were tested or evaluated for COVID-19 contraction, but this was not possible due to the large number of nurses and respiratory therapists providing care to ICU patients and the inability to have a dedicated group for the procedures.

In summary, this novel protocol is associated with a high success rate and appears to be safe for patients and healthcare providers. It is described in detail and is easy to implement.

Future larger and controlled studies to confirm these findings will be valuable.

## Figures and Tables

**Table 1:** Novel PDT protocol.

(1) Equipment and medication checklist prior to entering room
Disposable bronchoscope function check
Tracheostomy kit verification
Sedative and neuromuscular blocker drawing
(2) Universal protocol and time out outside the room followed by donning of augmented PPE (N95 and PAPR)
(3) Apnea trial before start to mimic apnea
100% FIO_2_ and current PEEP
Place ventilator on standby for 60 seconds
Pass test if able to tolerate without desaturations
(4) Administer sedatives and paralytics to minimize cough and movement
(5) Short apnea to add adaptor to circuit and insert bronchoscope in the ETT
(6) Pull back ETT while keeping cuff inflated
(7) Local anesthesia, incision, and insertion of introducer needle and angiocatheter by most experienced operator
(8)Place ventilator on standby and complete dilation and insertion of tracheostomy tube
(9) Connect ventilator to tracheostomy, inflate tracheostomy cuff, and resume ventilation. Verify inspiratory and expiratory volumes
(10) Remove the ETT and bronchoscope en bloc from oropharynx directly in a bag

PDT=percutaneous dilatational tracheostomy; PPE=personnel protective equipment; PAPR=powered air-purifying respirator; ETT=endotracheal tube.

**Table 2:** Clinical characteristics of COVID-19 patients undergoing PDT.

Variable	PDT patients (*n* = 32)
Age (y)	54 ± 12
Gender (male/female)	16/16
Body mass index (kg/m^2^)	33 ± 10 (min 17-max 66) 17 > 30
PCR positive at the time of PDT	20, 5 false negative
FIO_2_	44 ± 8 (min 30-max 70)
PEEP	8 ± 3 (min 5-max 14)
Anticoagulation at the time of PDT	24
ECMO at any time/ECMO at time of PDT	7/0
Days on ventilator before PDT	22 ± 8 (min 14-max 39)
Follow-up after PDT (d)	17 ± 10 (min 4-max 45)
Follow-up after initial intubation	40 ± 14 (min 22-max 72)
Outcome	
Weaned from MV	18 (56%)
Remain on MV	5 (16%)
Death	9 (28%)

## Data Availability

Data is available on a secure server in the Division of Pulmonary Medicine, Cooper University Hospital.
